# Thanks to Reviewers

**DOI:** 10.1002/tpg2.70275

**Published:** 2026-08-09

Maintaining the editorial standards of a scientific journal is the primary task of the journal editors. Their task is made much easier with the help of colleagues who are invited to review manuscripts. Through their critical comments and helpful suggestions, these volunteer reviewers have done much to maintain and further the quality of research reported in *The Plant Genome*. The members of *The Plant Genome* Journal Editorial Board express their appreciation to the following individuals who reviewed one or more manuscripts during the past year. This list might not be complete due to problems in collating such information from many sources. We extend our apologies to those reviewers whose names have not been included.

Abbai, Ragavendran

Abdullaziz, Sheidu

Abeyratne, Chanaka

Acquadro, Alberto

Acuna, Andrea

Adunola, Paul

Aduragbemi, Amo

Akinwale, Richard Olutayo

Alahmad, Samir

Altendorf, Kayla

Amas, Junrey

An, Xinmin

Anderson, James

Anumalla, Mahender

Aoun, Meriem

Arbelaez, Juan

Argenta, Jessica

Arra, Yugander

Aski, Muraleedhar S.

Atanda, Sikiru Adeniyi

Babar, Md. Ali

Babar, Saba

Babula‐Skowronska, Danuta

Baisakh, Niranjan

Bajgain, Prabin

Balakrishnan, Divya

Bao, Jinsong

Baraibar, Silvina

Bari, Md. Abdulla

Barmukh, Rutwik

Bassi, Filippo Maria

Baxter, Ivan

Bektas, Harun

Belaineh, Yemane Girma

Bélanger, Sébastien

Belzile, Francois

Ben Sadoun, Sarah

Bent, Andrew

Berraies, Samia

Bertolini, Edoardo

Best, Norman

Bhandari, Aditi

Bhattacharyya, Madan

Bheemanahalli, Raju

Bhering, Leonardo

Bilyeu, Kristin

Binelli, Giorgio

Bird, Kevin

Bohar, Rajaguru

Bollina, Venkatesh

Bombarely, Aureliano

Brault, Charlotte

Brunner, Stephanie

Bulle, Mallesham

Burt, Andrew

Cai, Xiwen

Campbell, Malachy

Cao, Qinghe

Cao, Yunpeng

Caraza‐Harter, Maria

Cattivelli, Luigi

Ceoloni, Carla

Chagne, David

Chakraborti,, Dr. Mridul

Chawla, Harmeet

Chen, Charles

Chen, Qiuyue

Chen, Senyu

Chen, Shisheng

Chen, Yinglong

Chen, Yu‐Run

Claessens, Annie

Clemente, Thomas

Clevenger, Josh

Cockerton, Helen

Colantonio, Vincent

Cooper, Mark

Costa, Rita

Crain, Jared

Creux, Nicky

Cui, Fa

da Silva Pereira, Guilherme

Dalid, Cheryl

Davies, John

Degu, Wuletaw Tadesse

Demirel, Ufuk

Devos, Katrien

DeWitt, Noah

Dias, Kaio

Diepenbrock, Christine

Dinglasan, Eric

Dixon, Laura

Dolezel, Jaroslav

Dong, Haixiao

Dorn, Kevin

Douches, David

Douchkov, Dimitar

Dreisigacker, Susanne

Duitama, Jorge

Earl, Hugh

Edger, Patrick

Endelman, Jeffrey

Enyew, Muluken

Escamilla Sanchez, Diana Marcela

Fabrizio, Jack

Fan, Kejing

Fang, Jun

Ferguson, Scott

Ferrão, Luis Felipe

Fetch, J. W. Mitchell

Fikere, Mulusew

Frary, Anne

Fritsche‐Neto, Roberto

Fuchs, Jörg

Fujimoto, Ryo

Gaiero, Paola

Gaire, Susmita

Gangurde, Sunil

Gano, Boubacar

Gao, Qingming

Garcia‐Abadillo, Julian

Ghimire, Bikash

Ghorbel, Mouna

Gill, Harsimardeep

Gimode, Winnie

Goel, Shailendra

Golisz‐Mocydlarz, Anna

Gorafi, Yasir

Gotarkar, Dhananjay

Gottschalk, Christopher

Gou, Xiangjian

Govindan, Velu

Grewal, Surbhi

Guan, Xueying

Guan, Yuefeng

Gudi, Santosh

Guo, Jia

Gururani, Mayank

Guyot, Romain

Ha, Jungmin

Halladakeri, Priyanka

Han, Guomin

Han, Linqian

Hanafi, Samira El

Hancock, Nathan

Hao, Ming

Haraldsson, Einar Baldvin

Hayes, Julie E.

He, Qiang

He, Shiyang

He, Xinyao

Heidari, Bahram

Henning, John

Hickok, David

Hoyos‐Villegas, Valerio

Hu, shengwei

Huang, Jinguang

Huang, Lin

Huang, Linkai

Huang, Lu

Huang, Xuehui

Huang, Yubi

Hudson, Matthew E.

Hudson, Owen

Hytoenen, Timo

Isidro Sanchez, Julio

Izquierdo, Paulo

Jarquin, Diego

Javed, Asim

Jayakodi, Murukarthick

Jiang, Jiming

Jiang, Shukun

Jiao, Chengzhi

Jordan, David

Jung, Michaela

Kahlon, Kaviraj

Kale, Sandip M

Kamel, Radwa

Kamphuis, Lars

Kang, Chunying

Kantar, Michael

Kapoor, Mahak

Karlström, Amanda

Kaur, Harpreet

Kaur, Lakhvir

Kausar, Mohd Adnan

Kebede, Aida

Khan, Aamir W.

Kim, Sung Ryul

Kissing Kucek, Lisa

Klos, Kathy

Kolkman, Judith

Kong, Fanjiang

Kopalli, Venkataramana

Kothandaraman, Harish

Kruppa, Klaudia

Kumar Saini, Dinesh

Kumar, Anil

Kumar, Anuj

Kumar, Rajendra

Kumar, Sundeep

Kunwar, Sudip

Kwon, Soon‐Wook

Le Guen, Vincent

Le Signor, Christine

Lee, Gwonjin

Lee, Keunsub

Lee, Seonghee

Legarra, Andres

Li, Aili

Li, Cong

Li, Guoliang

Li, Xuehui

Liang, Wanqi

Licciardello, Concetta

Lima, Dayane

Lin, Feng

Lin, Yu

Little, Chris

Liu, Bao

Liu, Beibei

Liu, Degao

Liu, Dengcai

Liu, Jianing

Liu, Kaiye

Liu, Sanzhen

Liu, Shaoshuai

Liu, Shuyu

Liu, Zhiyong

Locke, Anna

Lorenz, Aaron

Lozada, Dennis Nicuh

Lu, Jiawen

Lubanga, Nelson

Lyerly, Jeanette

Ma, Jianxin

Ma, Yu

Maalouf, Fouad

Macaya‐Sanz, David

Mahalingam, Ramamurthy

Mahmood, Tariq

Malmberg, Marie

Mankar, Sumeet

Mao, Long

Marla, Sandeep

Marone, Marina P.

Marston, Elliott

Mascher, Martin

Mason, Richard Esten

Mastrangelo, Anna Maria

Mauleon, Ramil

McBreen, Jordan

Melson, Ellen

Melzer, Rainer

Menicos, Debora

Mierek‐Adamska, Agnieszka

Misheva, Svetlana

Mondego, Jorge

Montoro, Pascal

Moose, Stephen

Morota, Gota

Muhammad Amir Gulzar, Rana

Mukherjee, Arup

Mura, Cameron

Murad Leite Andrade, Mario

Myers, Jim

Najafabadi, Mohsen Yoosefzadeh

Nascimento, Ana Carolina

Nascimento, Moyses

Negus, Karlene

Ni, Fei

Nicolao, Rodrigo

Nicolas, Philippe

Niu, Jishan

Onogi, Akio

Otwani, Daniel

Ozimati, Alfred

Paliwal, Rajneesh

Papin, Victor

Patel, Jinesh

Patterson, Eric

Pearson, Sofie

Peixoto, Marco

Perez‐Enciso, Miguel

Perez, Paulino

Petri, Cesar

Piepho, Hans‐Peter

Pino Del Carpio, Dunia

Pratas, Diogo

Pucker, Boas

Puglia, Giuseppe D.

Qi, Xiantao

Qiu, Changpeng

Rajan, S.

Rajcan, Istvan

Rani, Heena

Rao, Madhugiri

Rasheed, Awais

Raturi, Gaurav

Rauf, Yahya

Ravelombola, Waltram

Rawale, Kanwardeep

Rice, Brian

Rincent, Renaud

Robbins, Kelly

Robins, Joseph

Rock, Christopher

Rohila, Jai

Rooney, William

Roscher‐Ehrig, Lennard

Ross, Elizabeth

Rossini, laura

Roychowdhury, Rajib

Ruiz, Marcos

Sabadin, Felipe

Sagae, Vitor

Saini, Dinesh Kumar

Sakiroglu, Muhammet

Sakuma, Shun

Sallam, Ahmed

Salony, Susnata

Salvi, Silvio

Sandhu, Karansher Singh

Sandoval, Escoto

Sandoya, German

Sapkota, Manoj

Saripalli, Gautam

Sarup, Pernille

Schmickl, Roswitha

Schneider, Hannah

Schreiber, Miriam

Sekhon, Rajandeep

Sena, Saikat

Septiningsih, Endang

Shaffer, Will

Shahid, Muhammad Qasim

Shannon, Laura

Sharbel, Tim

Sharif, Yasir

Sharma, Ankush

Sharma, Pradeep

Sharma, Rajiv

Shellhammer, Tom

Shen, Qiufang

Shivaraj, S. M.

Shoeva, Olesya

Shrestha, Avinash

Shuijin, Hua

Singh, Deepti

Singh, Gurjeet

Singh, Jaswinder

Singh, Jugpreet

Singh, Nikhil Kumar

Singh, Vikas Kumar

Sinha, Pallavi

Sipowicz, Pablo

Skot, Leif

Smith, Kevin P.

Song, Qijian

Spanner, Rebecca

Spika, Ankica Kondic

Spychalla, Pia

Stack, George

Staton, Margaret

Steffenrem, Arne

Stoll, Hannah

Stoltz, Sophia

Stupar, Robert

Subedi, Madhav

Subudhi, Prasanta

Sun, Guangchao

Sun, Lei

Sun, Lianjun

Sun, Silong

Svara, Anze

Tai, Helen

Tamura, Ken‐ichi

Tan, Ek Han

Tan, Feng‐Quan

Tan, Kar‐Chun

Tanaka, Hidenori

Tanaka, Masaru

Taniguti, Cristiane

Tao, Dayun

Tehseen, Muhammad Massub

Thavarajah, Dil

Thomson, Susan

Thumma, Bala R.

Thuraga, Vishnukiran

Tian, Zhixi

Tiffin, Peter

Tiwari, Vijay

Tomura, Shunichiro

Tonk, Fatma

Toth, Jacob

Trubanová, Nina

Tsuchimatsu, Takashi

Tuberosa, Roberto

Turuspekov, Yerlan

Uhdre, Renan

Umer, Muhammad

Vaio, Magdalena

Valliyodan, Babu

Van der Laan, Liza

Vanous, Adam

Vergara, Georgina

Verma, Neha

Viboonjun, Unchera

Vieira, Caio

Vieira, Maria Lucia Carneiro

Villwock, Seren

Vitale, Paolo

Vleugels, Tim

Vogel, Gregory

Vuong, Tri

Wang, Fangyi

Wang, Jinmao

Wang, Lihao

Wang, lijun

Wang, Shaokui

Wang, Tian

Wang, Weidong

Wang, Xuewen

Wang, Yiling

Wang, Zhili

Warkentin, Tom

Wartha, Cleiton Antonio

Weber, Sven

Wei, Li

Weller, James

Weng, Jian Feng

Whitaker, Vance

Whitham, Steve

Wilkerson, Dustin

Wong, Landon

Wu, Xiang

Xavier, Alencar

Xie, Peng

Xin, Dawei

Xin, Liping

Xu, Fan

Xu, Gen

Xu, Xiangyang

Yadav, Shailesh

Yan, Jun

Yang, Chao

Yang, Shengming

Yang, Xinghai

Yang, Yuming

Yerka, Melinda

Yin, Dongmei

Yin, Tongming

Yol, Engin

Yu, Deyue

Yu, Jianming

Yu, Peng

Yuan, Guangsheng

Yusuf, Muyideen

Zhang, Chaozhong

Zhang, Dapeng

Zhang, Hengyou

Zhang, Huiting

Zhang, Jianwei

Zhang, Jing

Zhang, Junli

zhang, Wentao

Zhang, Xiaofei

Zhao, Peng

Zheng, Chaozhi

Zheng, Zhi

Zheng, Zhimin

Zheng, Zihao

Zhong, Tao

Zhong, Zhenhui

Zhou, Meixue

Zhou, Xun Bo

Zhou, Yan

Zhu, Jinjie

Zou, Zhi

